# Schrödinger Operators with Oblique Transmission Conditions in ${\mathbb R}^2$

**DOI:** 10.1007/s00220-023-04708-7

**Published:** 2023-05-02

**Authors:** Jussi Behrndt, Markus Holzmann, Georg Stenzel

**Affiliations:** Institut für Angewandte Mathematik, Technische Universität Graz, Steyrergasse 30, 8010 Graz, Austria

## Abstract

In this paper we study the spectrum of self-adjoint Schrödinger operators in $L^2(\mathbb {R}^2)$ with a new type of transmission conditions along a smooth closed curve $\Sigma \subseteq \mathbb {R}^2$. Although these *oblique* transmission conditions are formally similar to $\delta '$-conditions on $\Sigma$ (instead of the normal derivative here the Wirtinger derivative is used) the spectral properties are significantly different: it turns out that for attractive interaction strengths the discrete spectrum is always unbounded from below. Besides this unexpected spectral effect we also identify the essential spectrum, and we prove a Krein-type resolvent formula and a Birman-Schwinger principle. Furthermore, we show that these Schrödinger operators with oblique transmission conditions arise naturally as non-relativistic limits of Dirac operators with electrostatic and Lorentz scalar $\delta$-interactions justifying their usage as models in quantum mechanics.

## Introduction

In many quantum mechanical applications one considers particles moving in an external potential field which is localized near a set $\Sigma$ of measure zero. Such strongly localized fields can be modeled by singular potentials that are supported on $\Sigma$ only; of particular importance in this regard are $\delta$ and $\delta '$-interactions. To be more precise, assume that $\Sigma$ splits ${\mathbb R}^2$ into a bounded domain $\Omega _{+}$ and an unbounded domain $\Omega _{-} = {\mathbb R}^2 {\setminus } \overline{\Omega _{+}}$, and consider the formal Schrödinger differential expressions

1.1 $$\begin{aligned} \mathcal {H}_{\delta , \alpha } = - \Delta + \alpha \delta _{\Sigma } \quad \text {and} \quad \mathcal {H}_{\delta ', \alpha } = - \Delta + \alpha \delta '_{\Sigma },\quad \alpha \in {\mathbb R}. \end{aligned}$$

These singular perturbations of the free Schrödinger operator $-\Delta$ are characterized by certain transmission conditions along the interface $\Sigma$ for the functions in the operator domain. For $\delta$-interactions one considers functions $f: {\mathbb R}^2 \rightarrow {\mathbb C}$ such that the restrictions $f_{\pm } = f \upharpoonright \Omega _{\pm }$ satisfy the transmission conditions

1.2 $$\begin{aligned} f_{+} = f_{-} \quad \text {and} \quad -\frac{\alpha }{2} \big ( f_{+} + f_{-} \big ) = \big ( \partial _{\nu } f_{+} - \partial _{\nu } f_{-} \big ) \quad \text {on } \Sigma , \end{aligned}$$

while $\delta '$-interactions are modeled by the transmission conditions

1.3 $$\begin{aligned} f_{+} - f_{-} = -\frac{\alpha }{2 } \big ( \partial _{\nu } f_{+} + \partial _{\nu } f_{-} \big ) \quad \text {and} \quad \partial _{\nu } f_{+} = \partial _{\nu } f_{-} \quad \text {on } \Sigma ; \end{aligned}$$

here $\partial _{\nu } f_{\pm }$ is the normal derivative and $\nu = (\nu _1, \nu _2)$ the unit normal vector field on $\Sigma$ pointing outwards of $\Omega _{+}$. The spectra and resonances of the self-adjoint realizations associated with the formal expressions (1.1) in $L^2({\mathbb R}^2)$ are well understood, see, e.g., [8, 9, 12, 13, 15–18, 24]. In particular, the essential spectrum is given by $[0, \infty )$ and the discrete spectrum consists of at most finitely many points for every interaction strength $\alpha < 0$, while there is no negative spectrum if $\alpha \ge 0$.

In contrast to the transmission conditions (1.2) and (1.3) we are interested in a new type of transmission conditions of the form

1.4 $$\begin{aligned} (\nu _1 + i \nu _2) \big ( f_{+} - f_{-} \big ) = - \alpha \big ( \partial _{\overline{z}} f_{+} + \partial _{\overline{z}} f_{-} \big ) \quad \text {and} \quad \partial _{\overline{z}} f_{+} = \partial _{\overline{z}} f_{-} \quad \text {on } \Sigma , \end{aligned}$$

where $\alpha \in {\mathbb R}$ and $\partial _{\overline{z}} = \frac{1}{2} ( \partial _1 + i \partial _2)$ is the Wirtinger derivative. In the sequel such jump conditions will be referred to as *oblique* transmission conditions. Note that the conditions (1.4) can be rewritten as

1.5 $$\begin{aligned} f_{+} - f_{-} = - \frac{\alpha }{2} \big ( \partial _{\nu } f_{+} + \partial _{\nu } f_{-} + i \partial _{t} f_{+} + i \partial _{t} f_{-} \big ) \quad \text {and} \quad \partial _{\overline{z}} f_{+} = \partial _{\overline{z}} f_{-} \quad \text {on } \Sigma , \end{aligned}$$

where $\partial _t$ denotes the tangential derivative. Thus, on a formal level there is some analogy to the $\delta '$-transmission conditions in (1.3), but it will turn out that the properties of the corresponding self-adjoint realization in $L^2({\mathbb R}^2)$ differ significantly from those of Schrödinger operators with $\delta '$-interactions.

To make matters mathematically rigorous, assume that the curve $\Sigma$ is the boundary of a bounded and simply connected $C^\infty$-domain $\Omega _+$ with open complement $\Omega _{-} = {\mathbb R}^2 {\setminus } \overline{\Omega _{+}}$, denote the $L^2$-based Sobolev space of first order by $H^1$, let $\gamma _D^\pm :H^1(\Omega _{\pm })\rightarrow L^2(\Sigma )$ be the Dirichlet trace operators, and define for $\alpha \in {\mathbb R}$ the Schrödinger operator with oblique transmission conditions by

1.6 $$\begin{aligned} \begin{aligned} T_{\alpha } f&= \left( - \Delta f_{+} \right) \oplus \left( - \Delta f_{-} \right) ,\\ \mathrm{dom\,}T_{\alpha }&= \Big \{ f \in H^1(\Omega _{+}) \oplus H^1(\Omega _{-}) \, \big | \, \partial _{\overline{z}}f_{+} \oplus \partial _{\overline{z}} f_{-} \in H^1({\mathbb R}^2), \\& (\nu _1 + i \nu _2) \bigl (\gamma _D^+ f_{+} - \gamma _D^- f_{-} \bigr ) = - \alpha \bigl ( \gamma _D^+ ( \partial _{\overline{z}} f_{+}) + \gamma _D^- ( \partial _{\overline{z}} f_{-} )\bigr ) \Big \}. \end{aligned} \end{aligned}$$

The next theorem is the main result in this paper. We discuss the spectral properties of the Schrödinger operators $T_\alpha$ and, in particular, we show in item (ii) that for every $\alpha <0$ the operator $T_\alpha$ is necessarily unbounded from below and the discrete spectrum in $(-\infty ,0)$ is infinite and accumulates to $-\infty$. In items (iii) and (iv) we shall make use of the potential operator $\Psi _{\lambda }:L^2(\Sigma ) \rightarrow L^2({\mathbb R}^2)$ and the single layer boundary integral operator $S(\lambda ):L^2(\Sigma ) \rightarrow L^2(\Sigma )$ defined in (2.2) and (2.4), respectively.

### Theorem 1.1

For any $\alpha \in {\mathbb R}$ the operator $T_{\alpha }$ is self-adjoint in $L^2({\mathbb R}^2)$ and the essential spectrum is given by

$$\begin{aligned} \begin{aligned} \sigma _{\textrm{ess}}(T_{\alpha }) = [0, \infty ). \end{aligned} \end{aligned}$$

Furthermore, the following statements hold:

(i) If $\alpha \ge 0$, then $\sigma _{\textrm{disc}}(T_{\alpha }) = \emptyset$ and $T_\alpha$ is a nonnegative operator in $L^2({\mathbb R}^2)$.
(ii) If $\alpha < 0$, then $\sigma _{\textrm{disc}}(T_{\alpha })$ is infinite, unbounded from below, and does not accumulate to 0. Moreover, for every fixed $n \in \mathbb {N}$ the *n*-th discrete eigenvalue $\lambda _n \in \sigma _{\textrm{disc}}(T_{\alpha })$ (ordered non-increasingly) admits the asymptotic expansion
  $$\begin{aligned} \lambda _n = - \frac{4}{\alpha ^2} + \mathcal {O}(1) \quad \text {for } \alpha \rightarrow 0^{-}, \end{aligned}$$
  where the dependence on *n* appears in the $\mathcal {O}(1)$-term.
(iii) For $\lambda \in {\mathbb C}{\setminus } [0, \infty )$ the Birman-Schwinger principle is valid:
  $$\begin{aligned} \lambda \in \sigma _\textrm{p}(T_{\alpha }) \quad \Longleftrightarrow \quad 1 \in \sigma _\textrm{p} \big (\alpha \lambda S(\lambda ) \big ). \end{aligned}$$
(iv) For $\lambda \in \rho (T_{\alpha })={\mathbb C}{\setminus } ( [0, \infty ) \cup \sigma _\textrm{p}(T_{\alpha }))$ the operator $I - \alpha \lambda S(\lambda )$ is boundedly invertible in $L^2(\Sigma )$ and the resolvent formula
  $$\begin{aligned} (T_{\alpha } - \lambda )^{-1} = (- \Delta - \lambda )^{-1} +\alpha \Psi _{\lambda } \bigl ( I - \alpha \lambda S(\lambda ) \bigr )^{-1} \Psi _{\overline{\lambda }}^{*} \end{aligned}$$
  holds, where $- \Delta$ is the free Schrödinger operator defined on $H^2({\mathbb R}^2)$.

To illustrate the significance of Theorem 1.1 we show that Schrödinger operators with oblique transmission conditions arise naturally as non-relativistic limits of Dirac operators with electrostatic and Lorentz scalar $\delta$-interactions. To motivate this, consider one-dimensional Dirac operators with $\delta '$-interactions of strength $\alpha \in \mathbb {R}$ supported in the point $\Sigma =\{0\}$. These are first order differential operators in $L^2(\mathbb {R})^2$ and the singular interaction is modeled by transmission conditions for functions in the operator domain, which for sufficiently smooth $f = (f_1,f_2) \in L^2(\mathbb {R})^2$ are given by

1.7 $$\begin{aligned} f_1(0+)-f_1(0-) = i \frac{\alpha c}{2} \big ( f_2(0+)+f_2(0-) \big ) \quad \text {and} \quad f_2(0+) = f_2(0-), \end{aligned}$$

where $c>0$ is the speed of light. It is known that the associated self-adjoint Dirac operators converge in the non-relativistic limit to a Schrödinger operator with a $\delta '$-interaction of strength $\alpha$; cf. [2, 19] and also [10, 11] for generalizations. It is not difficult to see that (1.7) can be rewritten as the transmission conditions associated with a Dirac operator with a combination of an electrostatic and a Lorentz scalar $\delta$-interaction of strengths $\eta =- \frac{\alpha c^2}{2}$ and $\tau = \frac{\alpha c^2}{2}$, respectively, as they were studied in dimension one recently in [7] and in higher space dimensions in, e.g., [3, 5–7].

To find a counterpart of the above result in dimension two, consider a Dirac operator with electrostatic and Lorentz scalar $\delta$-shell interactions of strength $\eta$ and $\tau$, respectively, supported on $\Sigma$, which is formally given by

1.8 $$\begin{aligned} \mathcal {A}_{\eta , \tau } = A_0 + \left( \eta I_2 + \tau \sigma _3 \right) \delta _{\Sigma }; \end{aligned}$$

here $A_0$ is the unperturbed Dirac operator, $I_2$ is the $2 \times 2$-identity matrix and $\sigma _3 \in \mathbb {C}^{2 \times 2}$ is given in (3.1). The differential expression $\mathcal {A}_{\eta , \tau }$ gives rise to a self-adjoint operator $A_{\eta , \tau }$ in $L^2(\mathbb {R}^2)^2$, see (3.3). If one chooses, as above, $\eta = -\frac{\alpha c^2}{2}$ and $\tau = \frac{\alpha c^2}{2}$ and computes the non-relativistic limit, then instead of a Schrödinger operator with a $\delta '$-interaction one gets the somewhat unexpected limit $T_\alpha$. Of course, this is compatible with the one-dimensional result described above, as the one-dimensional counterparts of (1.3) and (1.5) coincide, since there are no tangential derivatives in $\mathbb {R}$. However, in higher dimensions Schrödinger operators with oblique transmission conditions should be viewed as the non-relativistic counterparts of Dirac operators with transmission conditions generalizing (1.7). Related results on non-relativistic limits of three-dimensional Dirac operators with singular interactions can be found in [4, 5, 21]. The precise result about the non-relativistic limit described above is stated in the following theorem and shown in Sect. 3.

### Theorem 1.2

Let $\alpha \in {\mathbb R}$. Then for all $\lambda \in \mathbb {C} {\setminus } \mathbb {R}$ one has

$$\begin{aligned} \lim _{c \rightarrow \infty } \left( A_{-\alpha c^2/2, \alpha c^2/2} - (\lambda + c^2 /2) \right) ^{-1} = \left( \begin{array}{cc} \left( T_{\alpha } - \lambda \right) ^{-1} &{} 0 \\ 0 &{} 0 \end{array} \right) , \end{aligned}$$

where the convergence is in the operator norm and the convergence rate is $\mathcal {O}\left( \frac{1}{c} \right)$.

**Notations** Throughout this paper $\Omega _{+} \subseteq {\mathbb R}^2$ is a bounded and simply connected $C^{\infty }$-domain and $\Omega _{-} = {\mathbb R}^2 {\setminus } \overline{\Omega _{+}}$ is the corresponding exterior domain with boundary $\Sigma = \partial \Omega _{-} = \partial \Omega _{+}$. The unit normal vector field on $\Sigma$ pointing outwards of $\Omega _+$ is denoted by $\nu$. Moreover, for $z \in {\mathbb C}{\setminus }[0, \infty )$ we choose the square root $\sqrt{z}$ such that $\text {Im} \sqrt{z} > 0$ holds. The modified Bessel function of order $j \in {\mathbb N}_0$ is denoted by $K_j$.

For $s \ge 0$ the spaces $H^s({\mathbb R}^2)^n$, $H^s(\Omega _{\pm })^n$, and $H^s(\Sigma )^n$ are the standard $L^2$-based Sobolev spaces of ${\mathbb C}^n$-valued functions defined on ${\mathbb R}^2$, $\Omega _{\pm }$, and $\Sigma$, respectively. If $n=1$ we simply write $H^s({\mathbb R}^2)$, $H^s(\Omega _{\pm })$, and $H^s(\Sigma )$. For negative $s < 0$ we define the spaces $H^s({\mathbb R}^2)^n$ and $H^s(\Sigma )^n$ as the anti-dual spaces of $H^{-s}({\mathbb R}^2)^n$ and $H^{-s}(\Sigma )^n$, respectively. We denote the restrictions of functions $f: \mathbb {R}^2 \rightarrow \mathbb {C}^n$ onto $\Omega _\pm$ by $f_\pm$; in this sense we write $H^1(\mathbb {R}^2 {{\setminus }} \Sigma )^n = H^1(\Omega _+)^n \oplus H^1(\Omega _-)^n$ and identify $f \in H^1(\mathbb {R}^2 {{\setminus }} \Sigma )^n$ with $f_+ \oplus f_-$, where $f_\pm \in H^1(\Omega _\pm )^n$. In the following $\gamma _D^\pm :H^1(\Omega _{\pm })\rightarrow L^2(\Sigma )$ denote the Dirichlet trace operators and we shall write $\gamma _D:H^1(\mathbb R^2)\rightarrow L^2(\Sigma )$ for the Dirichlet trace on $H^1(\mathbb R^2)$; sometimes these trace operators are also viewed as bounded mappings to $H^{1/2}(\Sigma )$.

For a Hilbert space $\mathcal {H}$ we write $\mathcal {L}(\mathcal {H})$ for the space of all everywhere defined, linear, and bounded operators on $\mathcal {H}$. Furthermore, the domain, kernel, and range of a linear operator *T* from a Hilbert space $\mathcal {G}$ to $\mathcal {H}$ are denoted by $\text {dom}\, T$, $\text {ker}\, T$, and $\text {ran}\, T$, respectively. The resolvent set, the spectrum, the essential spectrum, the discrete spectrum, and the point spectrum of a self-adjoint operator *T* are denoted by $\rho (T)$, $\sigma (T)$, $\sigma _{\text {ess}}(T)$, $\sigma _{\text {disc}}(T)$, and $\sigma _p (T)$. The eigenvalues of compact self-adjoint operators $K \in \mathcal {L}(\mathcal {H})$ are denoted by $\mu _n(K)$ and are ordered by their absolute values.

## Proof of Theorem 1.1

In this section the main result of this paper will be proved. For this, some families of integral operators are used. Define for $\lambda \in {\mathbb C}{\setminus } [0, \infty )$ the function $L_{\lambda }$ by

2.1 $$\begin{aligned} L_{\lambda }(x) = \frac{\sqrt{ \lambda }}{2 \pi } K_1 \bigl ( - i \sqrt{ \lambda } |x| \bigr ) \frac{ x_1 - i x_2}{|x|}, \quad x = (x_1,x_2) \in \mathbb {R}^2 {\setminus } \{0\}, \end{aligned}$$

and the operator $\Psi _{\lambda }: L^2(\Sigma ) \rightarrow L^2({\mathbb R}^2)$ by

2.2 $$\begin{aligned} \Psi _{\lambda } \varphi (x) = \int _{\Sigma } L_{\lambda }(x-y) \varphi (y) d \sigma (y), \quad \varphi \in L^2(\Sigma ), ~x \in {\mathbb R}^2{\setminus }\Sigma . \end{aligned}$$

Moreover, for $\lambda \in {\mathbb C}{\setminus } [0, \infty )$ we make use of the single layer potential $SL(\lambda ): L^2(\Sigma ) \rightarrow H^1(\mathbb {R}^2)$ and the single layer boundary integral operator $S(\lambda ): L^2(\Sigma ) \rightarrow L^2(\Sigma )$ associated with $-\Delta - \lambda$ that are defined by

2.3 $$\begin{aligned} SL(\lambda ) \varphi (x) = \int _\Sigma \frac{1}{2 \pi } K_0 \bigl ( - i \sqrt{\lambda } |x- y| \bigr ) \varphi (y) d \sigma (y), \quad \varphi \in L^2(\Sigma ),\quad x \in \mathbb {R}^2 {\setminus } \Sigma , \end{aligned}$$

and

2.4 $$\begin{aligned} S(\lambda ) \varphi (x) = \int _{\Sigma } \frac{1}{2 \pi } K_0 \bigl ( - i \sqrt{\lambda } |x- y| \bigr ) \varphi (y) d \sigma (y), \quad \varphi \in L^2(\Sigma ), ~x \in \Sigma . \end{aligned}$$

It is known that $SL(\lambda )$ and $S(\lambda )$ are bounded and $\text {ran}\, S(\lambda ) \subseteq H^{1}(\Sigma )$; cf. [25, Theorem 6.12 and Theorem 7.2]. In particular, $S(\lambda )$ gives rise to a compact operator in $H^s(\Sigma )$ for every $s \in [0,1]$. Furthermore, $S(\lambda )$ is self-adjoint and positive for $\lambda < 0$ (see *Step 1* in the proof of Proposition 2.2). Some properties of $\Psi _{\lambda }$ and $S(\lambda )$ that are important in the proof of Theorem 1.1 are summarized in the following two propositions; cf. Appendix A for the proof of Propositions 2.1 and 2.2.

### Proposition 2.1

Let $\lambda \in {\mathbb C}{\setminus } [0, \infty )$ and let $\Psi _{\lambda }$ be given by (2.2). Then

2.5 $$\begin{aligned} \Psi _{\lambda } = -2 i \partial _z SL(\lambda ): L^2(\Sigma ) \rightarrow L^2({\mathbb R}^2) \end{aligned}$$

is bounded and the following is true:

(i) $\Psi _{\lambda }$ gives rise to a bijective mapping $\Psi _{\lambda }: H^{1/2}(\Sigma ) \rightarrow \mathcal {H}_{\lambda }$, where
  $$\begin{aligned} \begin{aligned} \mathcal {H}_{\lambda }:= \big \{ f \in H^1(\mathbb {R}^2 {\setminus } \Sigma ) \, | \, \partial _{\overline{z}}f_{+} \oplus \partial _{\overline{z}} f_{-} \in H^1({\mathbb R}^2), \left( - \Delta - \lambda \right) f_{\pm } = 0\, \textrm{on }\, \Omega _{\pm } \big \}. \end{aligned} \end{aligned}$$
(ii) $\Psi _{\lambda }^{*}: L^2({\mathbb R}^2) \rightarrow L^2(\Sigma )$ is a compact operator, $\Psi _\lambda ^* = -2i \gamma _D \partial _{\overline{z}} (-\Delta -\overline{\lambda })^{-1}$, and $\mathrm{ran\,}\Psi _{\lambda }^{*} \subseteq H^{1/2}(\Sigma )$.
(iii) For all $\varphi \in H^{1/2}(\Sigma )$ the jump relations
  $$\begin{aligned} \begin{aligned} i ( \nu _1 + i \nu _2) \big ( \gamma _D^{+} ( \Psi _{\lambda } \varphi )_{+} - \gamma _D^{-} ( \Psi _{\lambda } \varphi )_{-} \big )&= \varphi ,\\ -i \big ( \gamma _D^{+} \partial _{\overline{z}} ( \Psi _{\lambda } \varphi )_{+} + \gamma _D^{-} \partial _{\overline{z}} ( \Psi _{\lambda } \varphi )_{-} \big )&= \lambda S(\lambda ) \varphi , \end{aligned} \end{aligned}$$
  hold.

For $\lambda < 0$ denote by $\mu _n(S(\lambda ))$ the discrete eigenvalues of the positive self-adjoint operator $S(\lambda )$ ordered non-increasingly and with multiplicities taken into account.

### Proposition 2.2

Let $S(\lambda )$ be defined by (2.4) and let $n \in \mathbb {N}$ be fixed. Then the following holds:

(i) The function $(- \infty , 0) \ni \lambda \mapsto \lambda \mu _n( S(\lambda ) )$ is continuous, strictly monotonically increasing and
  $$\begin{aligned} \lim _{\lambda \rightarrow 0^{-} }\lambda \mu _n( S(\lambda ) ) = 0 \quad \text {and} \quad \lim _{\lambda \rightarrow -\infty } \lambda \mu _n( S(\lambda ) ) = - \infty . \end{aligned}$$
(ii) For $a<0$ the unique solution $\lambda _n(a) \in (- \infty , 0)$ of $\lambda \mu _n(S(\lambda )) = a$ (see (i)) admits the asymptotic expansion $\lambda _n(a) = - 4 a^2 + \mathcal {O}(1)$ for $a \rightarrow - \infty$, where the dependence on *n* appears in the $\mathcal {O}(1)$-term.

### Proof of Theorem 1.1

*Step 1.* We verify that $T_{\alpha }$ is symmetric in $L^2({\mathbb R}^2)$. Observe first that for $f \in \mathrm{dom\,}T_\alpha$ we have $\partial _{\overline{z}} f_{\pm } \in H^1(\Omega _{\pm })$ and $\Delta f_\pm = 4 \partial _{z} \partial _{\overline{z}} f_{\pm } \in L^2(\Omega _{\pm })$, and hence $T_\alpha$ is well-defined. Moreover, as $C_0^{\infty }(\mathbb {R}^2 {{\setminus }} \Sigma ) \subseteq \mathrm{dom\,}T_{\alpha }$ it is also clear that $\text {dom}\,T_\alpha$ is dense. In order to show that $T_{\alpha }$ is symmetric, we note that integration by parts in $\Omega _{\pm }$ yields for $f,g \in \mathrm{dom\,}T_{\alpha }$

2.6 $$\begin{aligned} \begin{aligned}&( - \Delta f_{\pm } , g_{\pm } )_{L^2(\Omega _{\pm })} = ( -4 \partial _{z} \partial _{\overline{z}} f_{\pm } , g_{\pm } )_{L^2(\Omega _{\pm })} \\ {}&\qquad = 4 ( \partial _{\overline{z}} f_{\pm } , \partial _{\overline{z}} g_{\pm } )_{L^2(\Omega _{\pm })} \mp 2 \big ( (\nu _1 - i \nu _2) \gamma _D^{\pm } ( \partial _{\overline{z}} f_{\pm } ), \gamma _D^{\pm } g_{\pm } \big )_{L^2(\Sigma )} \\ {}&\qquad = 4 ( \partial _{\overline{z}} f_{\pm } , \partial _{\overline{z}} g_{\pm } )_{L^2(\Omega _{\pm })} \mp 2 \big ( \gamma _D^{\pm } ( \partial _{\overline{z}} f_{\pm }), (\nu _1 + i \nu _2) \gamma _D^{\pm } g_{\pm }) \big )_{L^2(\Sigma )}. \end{aligned} \end{aligned}$$

Now, consider (2.6) for $f=g$ and add the equations for $\Omega _{+}$ and $\Omega _{-}$. Then, using $\gamma _D^{+} ( \partial _{\overline{z}} f_{+})=\gamma _D^{-} ( \partial _{\overline{z}} f_{-})$ and the transmission condition for $f \in \mathrm{dom\,}T_{\alpha }$, one finds that

2.7 $$\begin{aligned} \begin{aligned} \big ( T_{\alpha } f , f \big )_{L^2({\mathbb R}^2)}&= 4 \big ( \Vert \partial _{\overline{z}} f_{+} \Vert ^2_{L^2(\Omega _{+})} + \Vert \partial _{\overline{z}} f_{-} \Vert ^2_{L^2(\Omega _{-})} \big ) \\ {}&\qquad - \big ( \gamma _D^{+} ( \partial _{\overline{z}} f_{+}) + \gamma _D^{-} ( \partial _{\overline{z}} f_{-} ) , (\nu _1 + i \nu _2) ( \gamma _D^{+} f_{+} - \gamma _D^{-} f_{-} ) \big )_{L^2(\Sigma )} \\ {}&= 4 \Vert \partial _{\overline{z}} f_{+} \oplus \partial _{\overline{z}} f_{-} \Vert ^2_{L^2(\mathbb {R}^2)} + \alpha \Vert \gamma _D^{+} ( \partial _{\overline{z}} f_{+}) + \gamma _D^{-} ( \partial _{\overline{z}} f_{-}) \Vert ^2_{L^2(\Sigma )} \in \mathbb {R}. \end{aligned} \end{aligned}$$

Since this holds for all $f \in \mathrm{dom\,}T_{\alpha }$, we conclude that $T_{\alpha }$ is symmetric.

*Step 2.* Proof of the Birman-Schwinger principle in (iii): To show the first implication, assume that $\lambda \in {\mathbb C}{\setminus } [0, \infty )$ with $1 \in \sigma _p ( \alpha \lambda S(\lambda ) )$ and choose $\varphi \in {\mathrm{ker\,}\,}( I - \alpha \lambda S(\lambda )) {\setminus } \{0\}$. Then it follows from the mapping properties of $S(\lambda )$ that $\varphi = \alpha \lambda S(\lambda ) \varphi \in H^{1/2}(\Sigma )$ holds. Therefore, Proposition 2.1 (i) implies that $f:= \Psi _{\lambda } \varphi \in \mathcal {H}_\lambda$ fulfils $f \ne 0$, $f \in H^1(\mathbb {R}^2 {\setminus } \Sigma )$, $\partial _{\overline{z}}f_{+} \oplus \partial _{\overline{z}} f_{-} \in H^1({\mathbb R}^2)$ and, as $\varphi \in {\mathrm{ker\,}\,}( 1 - \alpha \lambda S(\lambda )) {\setminus } \{0\}$, Proposition 2.1 (iii) implies

$$\begin{aligned} i (\nu _1 + i \nu _2) \big ( \gamma _D^{+} f_{+} - \gamma _D^{-} f_{-} \big ) = \varphi = \alpha \lambda S(\lambda ) \varphi = -i \alpha \big ( \gamma _D^{+} ( \partial _{\overline{z}} f_{+}) + \gamma _D^{-} ( \partial _{\overline{z}} f_{-} ) \big ). \end{aligned}$$

Hence, $f \in \mathrm{dom\,}T_\alpha$. Moreover, as $f \in \mathcal {H}_\lambda$, we conclude $f \in {\mathrm{ker\,}\,}( T_{\alpha } - \lambda ) {\setminus } \{0\}$ and hence $\lambda \in \sigma _p (T_{\alpha })$.

To show the second implication, we assume that $\lambda \in \sigma _p (T_{\alpha })$ is given and we choose $f \in {\mathrm{ker\,}\,}(T_{\alpha } - \lambda ) {\setminus } \{0\}$. Then, by Proposition 2.1 (i) there exists a unique $\varphi \in H^{1/2}(\Sigma )$ such that $f = \Psi _{\lambda } \varphi$. Moreover, using $f \in \mathrm{dom\,}T_\alpha$ and Proposition 2.1 (iii) one finds that

$$\begin{aligned} 0 = i (\nu _1 + i \nu _2) \big ( \gamma _D^{+} f_{+} - \gamma _D^{-} f_{-} \big ) + i \alpha \big ( \gamma _D^{+} ( \partial _{\overline{z}} f_{+}) + \gamma _D^{-} ( \partial _{\overline{z}} f_{-} ) \big ) = ( I - \alpha \lambda S(\lambda ))\varphi . \end{aligned}$$

Since $\varphi \ne 0$, we conclude $1 \in \sigma _p ( \alpha \lambda S(\lambda ))$.

*Step 3.* Next, we prove that $T_{\alpha }$ is a self-adjoint operator and the resolvent formula in (iv). Let $\lambda \in {\mathbb C}{\setminus } ( [0, \infty ) \cup \sigma _p (T_{\alpha }) )$ be fixed. First, we show that $I - \alpha \lambda S(\lambda )$ gives rise to a bijective map in $H^s(\Sigma )$ for every $s \in [0,1]$. Recall that $S(\lambda )$ is compact in $H^s(\Sigma )$. Since $I - \alpha \lambda S(\lambda )$ is injective for our choice of $\lambda$ by the Birman-Schwinger principle in (iii), Fredholm’s alternative shows that $I - \alpha \lambda S(\lambda )$ is indeed bijective.

Recall that $T_\alpha$ is symmetric; cf. *Step 1*. Hence, to show that $T_\alpha$ is self-adjoint, it suffices to verify that $\text {ran}(T_\alpha -\lambda ) = L^2(\mathbb {R}^2)$ holds for $\lambda \in {\mathbb C}{\setminus } ( [0, \infty ) \cup \sigma _p (T_{\alpha }) )$. Fix such a $\lambda$, let $f \in L^2(\mathbb {R}^2)$, and define

2.8 $$\begin{aligned} g = (- \Delta - \lambda )^{-1}f + \alpha \Psi _{\lambda } (I - \alpha \lambda S(\lambda ))^{-1} \Psi _{\overline{\lambda }}^{*} f, \end{aligned}$$

which is well-defined by the considerations above. Since $\Psi _{\overline{\lambda }}^{*} f \in H^{1/2}(\Sigma )$ by Proposition 2.1 (ii) and $(I - \alpha \lambda S(\lambda ))^{-1}$ is bijective in $H^{1/2}(\Sigma )$, we conclude with Proposition 2.1 (i) that $\Psi _{\lambda } (I - \alpha \lambda S(\lambda ))^{-1} \Psi _{\overline{\lambda }}^{*} f \in \mathcal {H}_\lambda \subseteq H^1(\mathbb {R}^2 {\setminus } \Sigma )$. In particular, with $(- \Delta - \lambda )^{-1}f \in H^2(\mathbb {R}^2)$ this implies that $g \in H^1(\mathbb {R}^2 {\setminus } \Sigma )$ and $\partial _{\overline{z}}g_{+} \oplus \partial _{\overline{z}} g_{-} \in H^1({\mathbb R}^2)$. Moreover, with Proposition 2.1(ii)–(iii) we obtain that

$$\begin{aligned} \begin{aligned}&i (\nu _1 + i \nu _2) \big ( \gamma _D^{+} g_{+} - \gamma _D^{-} g_{-} \big ) + i \alpha \big ( \gamma _D^{+} ( \partial _{\overline{z}}g_{+}) + \gamma _D^{-}(\partial _{\overline{z}} g_{-}) \big ) \\ {}&\qquad = \alpha (I - \alpha \lambda S(\lambda ) )^{-1} \Psi _{\overline{\lambda }}^{*} f - \alpha \Psi _{\overline{\lambda }}^{*} f - \alpha ^2 \lambda S(\lambda ) (I - \alpha \lambda S(\lambda ) )^{-1} \Psi _{\overline{\lambda }}^{*} f \\ {}&\qquad = \alpha (I - \alpha \lambda S(\lambda )) (I - \alpha \lambda S(\lambda ) )^{-1} \Psi _{\overline{\lambda }}^{*} f - \alpha \Psi _{\overline{\lambda }}^{*} f = 0 \end{aligned} \end{aligned}$$

and hence, $g \in \mathrm{dom\,}T_{\alpha }$. As $\Psi _{\lambda } (I - \alpha \lambda S(\lambda ))^{-1} \Psi _{\overline{\lambda }}^{*} f \in \mathcal {H}_\lambda$ by Proposition 2.1 (i), we conclude

$$\begin{aligned} \begin{aligned} \left( - \Delta - \lambda \right) g_{\pm }&= \left( -\Delta - \lambda \right) \big ( (- \Delta - \lambda )^{-1} f \big )_{\pm } + \alpha \left( - \Delta - \lambda \right) \big ( \Psi _{\lambda } (I - \alpha \lambda S(\lambda ) )^{-1} \Psi _{\overline{\lambda }}^{*} f \big )_{\pm }\\&= \left( - \Delta - \lambda \right) \big ( (- \Delta - \lambda )^{-1} f \big )_{\pm } = f_{\pm }, \end{aligned} \end{aligned}$$

i.e. $(T_{\alpha } - \lambda ) g = f$. Since $f \in L^2({\mathbb R}^2)$ was arbitrary, we conclude that $\mathrm{ran\,}(T_{\alpha } - \lambda ) = L^2({\mathbb R}^2)$ and that $T_{\alpha }$ is self-adjoint. Moreover, the resolvent formula in item (iv) follows from (2.8).

*Step 4.* Next, we show $\sigma _{\text {ess}}(T_{\alpha }) = [0, \infty )$. For this fix some $\lambda \in {\mathbb C}{\setminus } \mathbb {R}$. Since $\Psi _{\overline{\lambda }}^{*}: L^2({\mathbb R}^2) \rightarrow L^2(\Sigma )$ is compact by Proposition 2.1 (ii), the resolvent formula in (iv) implies that $(T_{\alpha } - \lambda )^{-1} - (-\Delta - \lambda )^{-1}$ is a compact operator in $L^2({\mathbb R}^2)$. Consequently, Weyl’s Theorem [27, Theorem XIII.14] yields that $\sigma _\text {ess}(T_\alpha ) = \sigma _\text {ess}(-\Delta )=[0,\infty )$.

*Step 5.* Proof of (i): Let $\alpha \ge 0$. Then, (2.7) implies that $T_\alpha$ is non-negative and hence, $\sigma (T_\alpha ) \subset [0,\infty )$. Since the latter set coincides with $\sigma _\text {ess}(T_\alpha )$, see *Step 4*, we conclude $\sigma _\text {disc}(T_\alpha )=\emptyset$.

*Step 6.* Proof of (ii): Let $\alpha <0$. Since $\sigma _{\text {ess}}(T_{\alpha }) = [0, \infty )$, it follows from the Birman-Schwinger principle in (iii) that

$$\begin{aligned} \sigma _{\text {disc}}(T_{\alpha }) = \left\{ \lambda _n \, | \, n \in {\mathbb N}\right\} = \left\{ \lambda < 0 \, | \, \exists n \in {\mathbb N}\text { such that } \lambda \mu _n(S(\lambda )) = \alpha ^{-1} \right\} \end{aligned}$$

holds. Note that by Proposition 2.2 the equation $\lambda \mu _n(S(\lambda )) = \alpha ^{-1}$ has a unique solution $\lambda _n$ for all $n \in \mathbb {N}$. Moreover, for any $n \in {\mathbb N}$ there cannot be infinitely many $k \ne n$ with $\lambda _n = \lambda _k$, since otherwise $\alpha ^{-1} < 0$ would be an eigenvalue with infinite multiplicity of the self-adjoint and compact operator $\lambda _n S(\lambda _n)$. Thus $\sigma _{\text {disc}}(T_{\alpha })$ is indeed an infinite set. Furthermore, as $S(\lambda )$ is a positive self-adjoint operator in $L^2(\Sigma )$; cf. *Step 1* in the proof of Proposition 2.2, we have by definition $\mu _n(S(\lambda )) \ge \mu _{n+1}(S(\lambda ))$ implying $\lambda \mu _n ( S(\lambda )) \le \lambda \mu _{n+1}(S(\lambda ))$. Therefore, the monotonicity of the map $\lambda \mapsto \lambda \mu _n (S(\lambda ))$ from Proposition 2.2 yields $\lambda _{n+1} \le \lambda _{n}$ for all $n \in {\mathbb N}$. This shows that 0 cannot be an accumulation point of the sequence $(\lambda _n)_{n \in {\mathbb N}}$ and as $\sigma _{\text {ess}}(T_{\alpha })\cap (-\infty ,0)=\emptyset$ the sequence $(\lambda _n)_{n \in {\mathbb N}}$ has no finite accumulation points, that is, $\sigma _{\text {disc}}(T_{\alpha })$ must be unbounded from below.

It remains to prove the asymptotic expansion in item (ii). By the above considerations $\lambda _n$ is determined as the unique solution of $\lambda \mu _n(S(\lambda )) = \alpha ^{-1}$. Clearly, if $\alpha \rightarrow 0^-$, then $a:= \alpha ^{-1} \rightarrow -\infty$. Hence, it follows from Proposition 2.2 (ii) with $a = \alpha ^{-1}$ that $\lambda _n = - \frac{4}{\alpha ^2} + \mathcal {O}(1)$ for $\alpha \rightarrow 0^-$ and that the dependence on *n* appears in the $\mathcal {O}(1)$-term. $\square$

## Proof of Theorem 1.2

In this section we show that $T_\alpha$ is the non-relativistic limit of a family of Dirac operators with electrostatic and Lorentz scalar $\delta$-shell potentials formally given by (1.8), whose interaction strengths are suitably scaled. First, we recall the rigorous definition of the operator $A_{\eta , \tau }$ associated with (1.8), see [5–7] for details. Let

3.1 $$\begin{aligned} \begin{aligned} \sigma _1 = \left( \begin{array}{cc} 0 &{}{} 1\\ 1 &{}{} 0 \\ \end{array}\right) , \sigma _2 = \left( \begin{array}{cc} 0 &{}{} -i\\ i &{}{} 0 \\ \end{array}\right) , \text{ and } \sigma _3 = \left( \begin{array}{cc} 1 &{}{} 0\\ 0 &{}{} -1 \\ \end{array}\right) \end{aligned} \end{aligned}$$

be the Pauli spin matrices and denote the $2 \times 2$ identity matrix by $I_2$. Furthermore, for $x = (x_1,x_2) \in {\mathbb R}^2$ we will use the abbreviations

3.2 $$\begin{aligned} \sigma \cdot x = \sigma _1 x_1 + \sigma _2 x_2 \quad \text {and} \quad \sigma \cdot \nabla = \sigma _1 \partial _1 + \sigma _2 \partial _2. \end{aligned}$$

We define Dirac operators with electrostatic and Lorentz scalar $\delta$-shell interactions of strengths $\eta , \tau \in {\mathbb R}$ in $L^2({\mathbb R}^2)^2$ by

3.3 $$\begin{aligned} \begin{aligned} A_{\eta , \tau } f&= \left( - i c (\sigma \cdot \nabla ) +\frac{c^2}{2} \sigma _3 \right) f_{+} \oplus \left( - i c (\sigma \cdot \nabla ) + \frac{c^2}{2} \sigma _3 \right) f_{-}, \\ \mathrm {dom\,}A_{\eta , \tau }&= \bigg \{ f \in H^1(\Omega _{+})^2 \oplus H^1(\Omega _{-})^2 \, \big | \\ {}&~~ i c (\sigma \cdot \nu ) \left( \gamma _D^{+}f_{+} - \gamma _D^{-}f_{-} \right) + \frac{1}{2} ( \eta I_2+ \tau \sigma _3 ) \left( \gamma _D^{+}f_{+} + \gamma _D^{-}f_{-} \right) = 0 \bigg \}. \end{aligned} \end{aligned}$$

It is shown in [6, 7] that $A_{\eta , \tau }$ is self-adjoint in $L^2({\mathbb R}^2)^2$, whenever $\eta ^2-\tau ^2 \ne 4 c^2$, and as in [5] one sees that these operators are the self-adjoint realisations of the formal differential expression (1.8). In the above definition we are using units such that $\hbar = 1$ and consider the mass $m=\frac{1}{2}$, but we keep the speed of light *c* as a parameter for the discussion of the non-relativistic limit $c \rightarrow \infty$.

Throughout this section we make use of the self-adjoint free Dirac operator $A_0$, which coincides with the operator$A_{0,0}$ given in (3.3) and which is defined on $H^1(\mathbb {R}^2)^2$. For $\lambda \in \rho (A_0)=\mathbb {C} {\setminus }\big ((-\infty , -\frac{c^2}{2}] \cup [\frac{c^2}{2}, \infty )\big )$ the integral kernel of the resolvent of $A_0$ is given by $G_\lambda (x-y)$, where $G_\lambda (x)$ is defined for $x \in {\mathbb R}^2 {\setminus } \{0\}$ by

3.4 $$\begin{aligned} G_{\lambda }(x)= & {} \frac{1}{2 \pi c} \sqrt{ \frac{\lambda ^2}{c^2} - \frac{c^2}{4}} K_1 \left( -i \sqrt{ \frac{\lambda ^2}{c^2} - \frac{c^2}{4}} |x| \right) \frac{1}{|x|} (\sigma \cdot x) \nonumber \\{} & {} + \frac{1}{2 \pi c} K_0 \left( -i \sqrt{ \frac{\lambda ^2}{c^2} - \frac{c^2}{4} } |x| \right) \left( \frac{\lambda }{c} I_2 + \frac{c}{2} \sigma _3 \right) ; \end{aligned}$$

cf. [6, equation (3.2)]. With this function we define the two families of integral operators

3.5 $$\begin{aligned} \begin{aligned} \Phi _{\lambda } \varphi (x)&= \int _{\Sigma } G_{\lambda }(x-y) \varphi (y) \textrm{d} \sigma (y), \quad \varphi \in L^2(\Sigma )^2, ~x \in {\mathbb R}^2{\setminus }\Sigma , \\ \mathcal {C}_{\lambda } \varphi (x)&= \lim _{\varepsilon \rightarrow 0^{+}} \int \limits _{\Sigma {\setminus } B(x,\varepsilon )} G_{\lambda }(x-y) \varphi (y) \textrm{d} \sigma (y), \quad \varphi \in L^2(\Sigma )^2, ~ x \in \Sigma , \end{aligned} \end{aligned}$$

where $B(x,\varepsilon )$ is the ball of radius $\varepsilon$ centered at *x*. Both operators $\Phi _{\lambda }: L^2(\Sigma )^2 \rightarrow L^2({\mathbb R}^2)^2$ and $\mathcal {C}_{\lambda }: L^2(\Sigma )^2 \rightarrow L^2(\Sigma )^2$ are well-defined and bounded; cf. [6, Proposition 3.3 and equation (3.7)].

In the following lemma, which is a preparation for the proof of Theorem 1.2, we will use the matrices

$$\begin{aligned} M_1 = \left( \begin{array}{cc} 1 &{} 0 \\ 0 &{} 0 \end{array} \right) , \quad M_2 = \left( \begin{array}{cc} 0 &{} 1 \\ 0 &{} 0 \end{array} \right) , \quad \text {and} \quad M_3 = \left( \begin{array}{cc} 0 &{} 0 \\ 0 &{} 1 \end{array} \right) ; \end{aligned}$$

products of scalar operators and matrices are understood componentwise, e.g.

$$\begin{aligned} (-\Delta - \lambda )^{-1} M_1 = \left( \begin{array}{cc} (-\Delta - \lambda )^{-1} &{} 0 \\ 0 &{} 0 \end{array} \right) : L^2({\mathbb R}^2)^2 \rightarrow L^2({\mathbb R}^2)^2. \end{aligned}$$

### Lemma 3.1

Let $\lambda \in {\mathbb C}{\setminus } {\mathbb R}$. Then there exists a constant $K>0$, depending only on $\lambda$ and $\Sigma$, such that the estimates

3.6a $$\begin{aligned}&\displaystyle \begin{aligned}&\displaystyle \big \Vert \big ( A_0 - (\lambda + c^2 / 2 ) \big ) ^{-1} - \left( - \Delta - \lambda \right) ^{-1} M_1 \big \Vert \le \frac{K}{c},&\end{aligned} \end{aligned}$$

3.6b $$\begin{aligned}&\displaystyle \big \Vert c \Phi _{\lambda + c^2 / 2} M_3 - \Psi _{\lambda } M_2 \big \Vert \le \frac{K}{c},&\end{aligned}$$

3.6c $$\begin{aligned}&\displaystyle \big \Vert c M_3 \Phi _{\lambda +c^2 / 2}^{*} - M_2^{\top } \Psi _{\lambda }^{*} \big \Vert \le \frac{K}{c},&\end{aligned}$$

3.6d $$\begin{aligned}&\displaystyle \big \Vert c^2 M_3 \mathcal {C}_{\lambda + c^2 / 2} M_3 - \lambda S(\lambda ) M_3 \big \Vert \le \frac{K}{c},&\end{aligned}$$

are valid for all sufficiently large $c>0$.

### Proof

We use a similar strategy as in the proof of [4, Proposition 5.2]. In the following let $\lambda \in {\mathbb C}{\setminus } {\mathbb R}$ be fixed. Then $\lambda + \frac{c^2}{2} \in {\mathbb C}{\setminus } {\mathbb R}$ and hence all operators in (3.6a)–(3.6d) are well-defined. One verifies by direct calculation that for sufficiently large $c>0$ and all $t \in [0,1]$

3.7 $$\begin{aligned} 0 < \frac{1}{2} \left| \sqrt{\lambda } \right| \le \left| \sqrt{ \lambda + t \frac{\lambda ^2}{c^2}} \right| \le \frac{3}{2} \left| \sqrt{ \lambda } \right| \quad \text {and} \quad \frac{1}{2} \text {Im} \sqrt{\lambda } \le \text {Im} \sqrt{ \lambda + t \frac{\lambda ^2}{c^2}} \end{aligned}$$

hold. With the well-known asymptotic expansions of the modified Bessel functions and $K_1'(z) = -K_0(z) - \frac{1}{z} K_1(z)$, (see [1]) one shows that there exist constants $\widehat{K}, \kappa , R > 0$, depending only on $\lambda$, such that

3.8 $$\begin{aligned} \left| K_j \left( - i \sqrt{ \lambda + t \frac{\lambda ^2}{c^2}} |x| \right) \right| \le \widehat{K} \left\{ \begin{array}{ll} |x|^{-1}, &{} \text {for } |x| < R, \\ e^{- \kappa |x|}, &{} \text {for } |x| \ge R, \end{array}\right. \end{aligned}$$

and

3.9 $$\begin{aligned} \left| K_1' \left( - i \sqrt{ \lambda + t \frac{\lambda ^2}{c^2}} |x| \right) \right| \le \widehat{K} \left\{ \begin{array}{ll} |x|^{-2}, &{} \text {for } |x| < R, \\ e^{- \kappa |x|}, &{} \text {for } |x| \ge R, \end{array}\right. \end{aligned}$$

hold for all $x \in {\mathbb R}^2 {\setminus } \{0\}$, $j \in \{0,1\}$, $t \in [0,1]$, and sufficiently large $c>0$.

Next, with $G_{\lambda +c^2/2}$ defined by (3.4) we find

3.10 $$\begin{aligned} G_{\lambda + c^2 / 2 }(x)= & {} \frac{1}{2\pi c} \sqrt{\lambda +\frac{\lambda ^2}{c^2}} K_1 \left( - i \sqrt{\lambda +\frac{\lambda ^2}{c^2}} |x| \right) \frac{1}{|x|} ( \sigma \cdot x) \nonumber \\{} & {} + \frac{1}{2 \pi c} K_0 \left( -i \sqrt{\lambda +\frac{\lambda ^2}{c^2}} |x| \right) \left( \frac{\lambda }{c} I_2 + c M_1 \right) . \end{aligned}$$

Let

$$\begin{aligned} U_\lambda (x) = \frac{1}{2 \pi } K_0 \bigl ( - i \sqrt{ \lambda } |x| \bigr ), \quad x \in {\mathbb R}^2 {\setminus } \{0\}, \end{aligned}$$

be the integral kernel of the resolvent of the free Laplace operator; cf. [28, Chapter 7.5]. Then

$$\begin{aligned} G_{\lambda + c^2 / 2}(x) - U_\lambda (x) M_1 = t_1(x) + t_2(x) + t_3(x) \end{aligned}$$

holds, where the matrix-valued functions $t_1, t_2$, and $t_3$ are given by

$$\begin{aligned} \begin{aligned} t_1(x)&= \frac{1}{2 \pi c} \sqrt{ \lambda + \frac{\lambda ^2}{c^2}} K_1 \left( -i \sqrt{ \lambda + \frac{\lambda ^2}{c^2}} |x| \right) \frac{ \sigma \cdot x}{|x|}, \\ t_2(x)&= \frac{1}{2 \pi } \left( K_0 \left( -i \sqrt{ \lambda + \frac{\lambda ^2}{c^2}} |x| \right) - K_0 \bigl ( - i \sqrt{ \lambda } |x| \bigr ) \right) M_1, \\ t_3(x)&= \frac{\lambda }{2 \pi c^2} K_0 \left( -i \sqrt{ \lambda + \frac{\lambda ^2}{c^2}} |x| \right) I_2. \end{aligned} \end{aligned}$$

With (3.7) and (3.8) applied with $t=1$ one finds that there exist constants $k_1, \kappa , R > 0$, depending only on $\lambda$, such that for $j \in \{1,3\}$ and sufficiently large $c>0$ one has

$$\begin{aligned} \left| t_j(x) \right| \le \frac{k_1}{c} \left\{ \begin{array}{ll} |x|^{-1}, &{} \text {for } |x| < R, \\ e^{- \kappa |x|}, &{} \text {for } |x| \ge R. \end{array}\right. \end{aligned}$$

To estimate $t_2$, we use $K_0' = -K_1$ and obtain with the fundamental theorem of calculus, (3.7), and (3.8)

3.11 $$\begin{aligned} \begin{aligned}&\left| K_0 \left( -i \sqrt{ \lambda + \frac{\lambda ^2}{c^2}} |x| \right) - K_0 \left( - i \sqrt{ \lambda } |x| \right) \right| \le \int _0^1 \left| \frac{d}{d t} K_0 \left( -i \sqrt{ \lambda + t \frac{\lambda ^2}{c^2}} |x| \right) \right| d t \\ {}&\quad = \int _0^1 \frac{|\lambda |^2 |x|}{\left| \sqrt{ \lambda + t \frac{\lambda ^2}{c^2}} \right| } \frac{1}{2 c^2} \left| K_1 \left( -i \sqrt{ \lambda + t \frac{\lambda ^2}{c^2}} |x| \right) \right| d t \\ {}&\quad \le \frac{k_2}{c^2} \left\{ \begin{array}{ll} 1, &{}{} \text{ for } |x| < R, \\ e^{- \frac{\kappa }{2} |x|}, &{}{} \text{ for } |x| \ge R, \end{array}\right. \end{aligned} \end{aligned}$$

with a constant $k_2$ which depends only on $\lambda$. Thus, if we define $k_3 = 2 k_1 + \frac{k_2 R}{2 \pi }$, then

$$\begin{aligned} \left| G_{\lambda + c^2 / 2}(x) - U_\lambda (x) M_1 \right| \le \frac{k_3}{c} \left\{ \begin{array}{ll} |x|^{-1}, &{} \text {for } |x| < R, \\ e^{- \frac{\kappa }{2} |x|}, &{} \text {for } |x| \ge R. \end{array}\right. \end{aligned}$$

This estimation for the integral kernel yields with the Schur test; cf. [4, Proposition A.3] for a similar argument,

$$\begin{aligned} \begin{aligned} \big \Vert \big ( A_0 - (\lambda + c^2 / 2) \big ) ^{-1} - \left( -\Delta - \lambda \right) ^{-1} M_1 \big \Vert \le \frac{K}{c} \end{aligned} \end{aligned}$$

for all sufficiently large $c>0$, which is the first claimed estimate (3.6a).

Next, we prove (3.6b). Recall that the integral kernel $L_{\lambda }$ of $\Psi _\lambda$ is given by (2.1). Using that $\sigma _1 M_3 = M_2$, $\sigma _2 M_3 = -i M_2$, and $M_1 M_3 = 0$, we obtain with (3.10) the decomposition

$$\begin{aligned} c G_{\lambda + c^2 / 2}(x) M_3 - L_{\lambda } (x) M_2 = \tau _1(x) + \tau _2(x) + \tau _3(x) \end{aligned}$$

with

$$\begin{aligned} \begin{aligned} \tau _1(x)&= \frac{1}{2 \pi } \left( \sqrt{ \lambda + \frac{\lambda ^2}{c^2}} - \sqrt{\lambda } \right) K_1 \left( -i \sqrt{ \lambda + \frac{\lambda ^2}{c^2}} |x| \right) \frac{x_1 - i x_2}{|x|} M_2, \\ \tau _2(x)&= \frac{\sqrt{ \lambda }}{2 \pi } \left( K_1 \left( -i \sqrt{ \lambda + \frac{\lambda ^2}{c^2}} |x| \right) - K_1 \bigl ( -i \sqrt{ \lambda } |x| \bigr ) \right) \frac{x_1 - i x_2}{|x|} M_2, \\ \tau _3(x)&= \frac{\lambda }{2 \pi c} K_0 \left( -i \sqrt{ \lambda + \frac{\lambda ^2}{c^2}} |x| \right) M_3. \end{aligned} \end{aligned}$$

Similar as above it can be shown that there exists a $k_4>0$, depending only on $\lambda$, such that for all $j \in \{1,2,3\}$

$$\begin{aligned} \left| \tau _j(x) \right| \le \frac{k_4}{c} \left\{ \begin{array}{ll} |x|^{-1}, &{} \text {for } |x| < R, \\ e^{- \frac{\kappa }{2} |x|}, &{} \text {for } |x| \ge R; \end{array}\right. \end{aligned}$$

to see the estimate for $\tau _2$ one has to use (3.9). With the help of the Schur test the estimate (3.6b) follows (see also [4, Proposition A.4] for a similar argument); the constant $k_4$ depends in this case on $\lambda$ and $\Sigma$. The estimate in (3.6c) follows by taking adjoints.

It remains to prove (3.6d). Taking $M_3 (\sigma \cdot x) M_3 = 0$, which holds for any $x \in \mathbb {R}^2$, and (3.11) into account we obtain that

$$\begin{aligned} \begin{aligned}&\big | c^2 M_3 G_{\lambda + c^2 / 2}(x) M_3 - \lambda U_\lambda (x) M_3 \big | \\&\quad = \frac{|\lambda |}{2 \pi } \left| K_0 \left( -i \sqrt{ \lambda + \frac{\lambda ^2}{c^2}} |x| \right) - K_0 \bigl ( -i \sqrt{ \lambda } |x| \bigr ) \right| \le \frac{k_5}{c^2} \end{aligned} \end{aligned}$$

holds for all $x \in {\mathbb R}^2 {\setminus } \{0\}$. Using the dominated convergence theorem, one sees that

$$\begin{aligned} \big ( c^2 M_3 \mathcal {C}_{\lambda + c^2 / 2} M_3 f \big )(x) = \int _{\Sigma } c^2 M_3 G_{\lambda + c^2 / 2}(x-y) M_3 f(y) d \sigma (y) \end{aligned}$$

holds for all $f \in L^2(\Sigma )^2$ and $x \in \Sigma$, i.e. the integral does not have to be understood as principal value. Thus we obtain with the Schur test [23, III. Example 2.4] that

$$\begin{aligned} \begin{aligned} \big \Vert c^2 M_3 \mathcal {C}_{\lambda + m c^2} M_3 - \lambda S(\lambda ) M_3 \big \Vert \le \frac{K}{c^2}. \end{aligned} \end{aligned}$$

In this case, the constant *K* depends on $\lambda$ and $\Sigma$. This yields (3.6d) and finishes the proof of this lemma. $\square$

Now we are prepared to prove Theorem 1.2 and show that $A_{-\alpha c^2/2, \alpha c^2/2}$ converges in the non-relativistic limit to $T_\alpha$ defined in (1.6).

### Proof of Theorem 1.2

Let $\lambda \in {\mathbb C}{\setminus } {\mathbb R}$ be fixed. Then it follows from [7, Lemma 5.4, Proposition 5.5, Theorem 5.6, and Lemma 5.9] (see also [6, Theorem 4.6]) that the operator $I_2 - \alpha c^2 M_3 \mathcal {C}_{\lambda + c^2/2}: L^2(\Sigma )^2 \rightarrow L^2(\Sigma )^2$ is boundedly invertible and the resolvent of $A_{- \alpha c^2/2, \alpha c^2/2} - c^2/2$ is given by

3.12 $$\begin{aligned} \begin{aligned}\big (A_{-\alpha c^2/2 , \alpha c^2/2} -&(\lambda + c^2/2) \big )^{-1} = \big ( A_{0}-(\lambda +c^2/2)\big ) ^{-1}\\ {}&+ \Phi _{\lambda + c^2/2} \left( I - \alpha c^2 M_3 \mathcal {C}_{\lambda + c^2/2} \right) ^{-1} \alpha c^2 M_3 \Phi _{\overline{\lambda } + c^2/2}^{*}. \end{aligned} \end{aligned}$$

Because of $M_3 = M_3^2$ it follows from [26, Proposition 2.1.8] that

$$\begin{aligned} \sigma \big ( M_3 \mathcal {C}_{\lambda +c^2/2} \big ) \cup \{ 0 \} = \sigma \big ( M_3 \mathcal {C}_{\lambda + c^2 / 2} M_3 \big ) \cup \{ 0 \}. \end{aligned}$$

In particular, this yields that the operator $I - \alpha c^2 M_3 \mathcal {C}_{\lambda + c^2/2} M_3$ is boundedly invertible in $L^2(\Sigma )^2$ for all $c>0$ and a direct calculation shows

3.13 $$\begin{aligned} (I - \alpha c^2 M_3 \mathcal {C}_{\lambda + c^2 / 2} )^{-1} M_3 = M_3 ( I - \alpha c^2 M_3 \mathcal {C}_{\lambda + c^2 / 2} M_3)^{-1}. \end{aligned}$$

Recall that for $\lambda \in \mathbb {C} {\setminus } \mathbb {R}$ also $I - \alpha \lambda S(\lambda )$ is boundedly invertible in $L^2(\Sigma )$; cf. Theorem 1.1 (iv). Hence, we obtain from Lemma 3.1 and [23, IV. Theorem 1.16] that

3.14 $$\begin{aligned} \begin{aligned} \big \Vert ( I - \alpha c^2 M_3 \mathcal {C}_{\lambda + c^2 / 2} M_3)^{-1} - (I - \alpha \lambda S(\lambda ) M_3)^{-1} \big \Vert \le \frac{K}{c} \end{aligned} \end{aligned}$$

holds for all sufficiently large $c>0$ with a constant $K>0$ which depends only on $\lambda$, $\alpha$, and $\Sigma$.

To conclude, note that (3.12) and (3.13) yield

$$\begin{aligned} \begin{aligned} \big (A_{-\alpha c^2/2 , \alpha c^2/2} -&(\lambda + c^2 / 2) \big )^{-1} = \big ( A_0 - (\lambda + c^2 / 2) \big )^{-1} \\&+ c \Phi _{\lambda + c^2 / 2} M_3 ( I - \alpha c^2 M_3 \mathcal {C}_{\lambda + c^2 / 2} M_3)^{-1} \alpha c M_3 \Phi _{\overline{\lambda }+ c^2/2} ^{*}, \end{aligned} \end{aligned}$$

while Theorem 1.1 (iv) and $M_2 M_3 M_2^{\top } = M_1$ show

$$\begin{aligned} \begin{aligned} (T_{\alpha } - \lambda )^{-1} M_1&= ( - \Delta - \lambda )^{-1} M_1 + \Psi _{\lambda } ( I - \alpha \lambda S(\lambda ) )^{-1} \alpha \Psi _{\overline{\lambda }}^{*} M_1 \\&= ( - \Delta - \lambda )^{-1} M_1 + \Psi _{\lambda } M_2 (I - \alpha \lambda S(\lambda ) M_3)^{-1} \alpha M_2^{\top } \Psi _{\overline{\lambda }}^{*}. \end{aligned} \end{aligned}$$

Using Lemma 3.1 and (3.14) the last two displayed formulae finally lead to the claimed convergence result and it also follows that the order of convergence is $\mathcal {O}(\frac{1}{c})$. $\square$

## Proof of Propositions 2.1 and 2.2

Recall that for $\lambda \in \mathbb {C} {\setminus } [0, \infty )$ the operators $\Psi _\lambda$, $SL(\lambda )$, and $S(\lambda )$ are defined by (2.2), (2.3), and (2.4), respectively. First, we collect some properties of the single layer potential $SL(\lambda )$ that are needed in the following. It is well-known that $SL(\lambda ): H^{1/2}(\Sigma ) \rightarrow H^2(\mathbb {R}^2 {\setminus } \Sigma )$ gives rise to a bounded operator, that $(-\Delta - \lambda ) SL(\lambda ) \varphi =0$ in $\mathbb {R}^2 {\setminus } \Sigma$, and that for $\varphi \in H^{1/2}(\Sigma )$ the jump relations

A.1 $$\begin{aligned} \gamma _D^+ (SL(\lambda ) \varphi )_{+} = \gamma _D^- (SL(\lambda ) \varphi )_{-} \quad \text {and} \quad \partial _{\nu } (SL(\lambda ) \varphi )_{+} - \partial _{\nu } (SL(\lambda ) \varphi )_{-} = \varphi \end{aligned}$$

hold; cf. [25] or [22, Section 3.3]. Furthermore, for the single layer boundary integral operator $S(\lambda )$ from (2.4) we have $S(\lambda ) = \gamma _D SL(\lambda )$ and for all $\varphi \in L^2(\Sigma )$ the representations

A.2 $$\begin{aligned} SL(\lambda ) \varphi = (-\Delta - \lambda )^{-1} \gamma _D' \varphi \quad \text {and} \quad S(\lambda ) \varphi = \gamma _D (-\Delta - \lambda )^{-1} \gamma _D' \varphi \end{aligned}$$

hold (see [22, 25]); here $\gamma _D:H^1(\mathbb R^2)\rightarrow L^2(\Sigma )$ and $\gamma _D':L^2(\Sigma )\rightarrow H^{-1}(\mathbb R^2)$ is the anti-dual operator.

### Proof of Proposition 2.1

First, we prove item (ii). For $\lambda \in {\mathbb C}{\setminus } [0, \infty )$ define the operator

A.3 $$\begin{aligned} \widehat{\Psi }_\lambda := -2i \gamma _D \partial _{\overline{z}} (-\Delta -\overline{\lambda })^{-1}. \end{aligned}$$

Since $(-\Delta -\overline{\lambda })^{-1}: L^2(\mathbb {R}^2) \rightarrow H^2(\mathbb {R}^2)$ and $\gamma _D: H^1(\mathbb {R}^2) \rightarrow H^{1/2}(\Sigma )$ are bounded, we get that $\widehat{\Psi }_{\lambda }: L^2({\mathbb R}^2) \rightarrow H^{1/2}(\Sigma )$ is well-defined and bounded. Furthermore, as $H^{1/2}(\Sigma )$ is compactly embedded in $L^2(\Sigma )$ by Rellich’s embedding theorem, the operator $\widehat{\Psi }_{\lambda }: L^2({\mathbb R}^2) \rightarrow L^2(\Sigma )$ is compact. Note that $\widehat{\Psi }_\lambda$ is an integral operator with integral kernel

$$\begin{aligned} \begin{aligned} k(x,y)&= -2i \partial _{\overline{z}} \frac{1}{2 \pi } K_0 \Bigl ( - i \sqrt{\overline{\lambda }} |x- y| \Bigr ) \\ {}&= \frac{\sqrt{ \overline{\lambda }}}{2 \pi } K_1 \Bigl ( - i \sqrt{ \overline{\lambda }} |x-y| \Bigr ) \frac{ x_1-y_1 + i (x_2-y_2)}{|x-y|} \\ {}&= \overline{L_\lambda (y-x)}, \end{aligned} \end{aligned}$$

where we used $K_0'=-K_1$ in the second step and $\overline{\sqrt{\lambda }} = - \sqrt{\overline{\lambda }}$ in the last step (recall that $Im \sqrt{\omega } > 0$ for $\omega \in \mathbb {C} {\setminus } [0, \infty )$). Hence, we conclude that

$$\begin{aligned} \Psi _\lambda = \widehat{\Psi }_\lambda ^*: L^2(\Sigma ) \rightarrow L^2(\mathbb {R}^2) \end{aligned}$$

is bounded and that all claims in item (ii) are true.

Next, we show (2.5). Let $\varphi \in L^2(\Sigma )$ and $f \in H^1(\mathbb {R}^2)$. Since $\Delta = 4 \partial _{\overline{z}} \partial _z = 4 \partial _z \partial _{\overline{z}}$, we see that $\partial _{\overline{z}} (-\Delta -\overline{\lambda })^{-1} f = (-\Delta -\overline{\lambda })^{-1} \partial _{\overline{z}} f$. Hence, item (ii) and (A.2) imply

$$\begin{aligned} \begin{aligned} (\Psi _\lambda \varphi , f)_{L^2(\Sigma )}&= \big (\varphi , -2i \gamma _D \partial _{\overline{z}} (-\Delta -\overline{\lambda })^{-1} f\big )_{L^2(\mathbb {R}^2)} \\&=\big (\varphi , -2i \gamma _D (-\Delta -\overline{\lambda })^{-1} \partial _{\overline{z}} f\big )_{L^2(\mathbb {R}^2)} \\&= \big (-2 i \partial _z SL(\lambda ) \varphi , f\big )_{L^2(\mathbb {R}^2)}. \end{aligned} \end{aligned}$$

Since $H^1(\mathbb {R}^2)$ is dense in $L^2(\mathbb {R}^2)$, we conclude that (2.5) is true. In particular, this and the properties of the single layer potential mentioned at the beginning of this appendix imply that

A.4 $$\begin{aligned} \Psi _\lambda : H^{1/2}(\Sigma ) \rightarrow H^1(\mathbb {R}^2 {\setminus } \Sigma ) \end{aligned}$$

is bounded and for $\varphi \in H^{1/2}(\Sigma )$ we have

A.5 $$\begin{aligned} i \partial _{ \overline{z}} \left( \Psi _{\lambda } \varphi \right) _{\pm } = 2 \partial _{\overline{z}} \partial _{z} \left( SL(\lambda ) \varphi \right) _{\pm } = \frac{1}{2} \Delta \left( SL(\lambda ) \varphi \right) _{\pm } = - \frac{\lambda }{2} \left( SL(\lambda ) \varphi \right) _{\pm }. \end{aligned}$$

Since $SL(\lambda ) \varphi \in H^1({\mathbb R}^2)$ it follows that $\partial _{ \overline{z}} \left( \Psi _{\lambda } \varphi \right) _{+} \oplus \partial _{ \overline{z}} \left( \Psi _{\lambda } \varphi \right) _{-} \in H^1({\mathbb R}^2)$ holds for any $\varphi \in H^{1/2}(\Sigma )$.

Now, we show (iii). Let $\varphi \in H^{1/2}(\Sigma )$. With (A.5) we see that

$$\begin{aligned} \begin{aligned} -i \left( \gamma _D^+ \partial _{\overline{z}} ( \Psi _{\lambda } \varphi )_{+} + \gamma _D^- \partial _{\overline{z}} ( \Psi _{\lambda } \varphi )_{-}\right) = \lambda \gamma _D SL(\lambda ) \varphi = \lambda S(\lambda ) \varphi \end{aligned} \end{aligned}$$

holds. Moreover, we obtain with $SL(\lambda ) \varphi \in H^2(\mathbb {R}^2 {\setminus } \Sigma )$

$$\begin{aligned} i ( \nu _1 + i \nu _2) \gamma ^{\pm }_D \left( -2 i \partial _z SL(\lambda ) \varphi \right) _{\pm } = \partial _\nu \left( SL(\lambda ) \varphi \right) _{\pm } - i \partial _t \left( SL(\lambda ) \varphi \right) _{\pm }, \end{aligned}$$

where $\partial _t$ is the tangential derivative on $\Sigma$. As $SL(\lambda ) \varphi \in H^1(\mathbb {R}^2)$, one has the relation $\partial _t\left( SL(\lambda ) \varphi \right) _{+} = \partial _t \left( SL(\lambda ) \varphi \right) _{-}$ and consequently with (A.1)

$$\begin{aligned} i ( \nu _1 + i \nu _2) \left( \gamma ^{+}_D ( \Psi _{\lambda } \varphi )_{+} - \gamma ^{-}_D ( \Psi _{\lambda } \varphi )_{-} \right) = \partial _\nu \left( SL(\lambda ) \varphi \right) _{+} - \partial _\nu \left( SL(\lambda ) \varphi \right) _{-} = \varphi . \end{aligned}$$

This finishes the proof of (iii).

It remains to prove item (i). By applying the Wirtinger derivative $\partial _{z}$ to (A.5) one gets with (2.5) that

$$\begin{aligned} -\Delta \left( \Psi _{\lambda } \varphi \right) _{\pm } = -4 \partial _{z} \partial _{\overline{z}} \left( \Psi _{\lambda } \varphi \right) _{\pm } = -2 i \lambda \partial _{z} \left( SL(\lambda ) \varphi \right) _{\pm } = \lambda \left( \Psi _{\lambda } \varphi \right) _{\pm } \end{aligned}$$

holds for all $\varphi \in L^2(\Sigma )$ in the distributional sense. This, (A.4), (A.5), and the properties of $SL(\lambda )$ described at the beginning of this appendix show that $\Psi _{\lambda } \varphi \in \mathcal {H}_{\lambda }$ for all $\varphi \in H^{1/2}(\Sigma )$ and therefore the mapping $\Psi _{\lambda }: H^{1/2}(\Sigma ) \rightarrow \mathcal {H}_{\lambda }$ is well-defined. Moreover, it follows from (iii) that this mapping is injective. To prove that $\Psi _{\lambda }: H^{1/2}(\Sigma ) \rightarrow \mathcal {H}_{\lambda }$ is surjective, let $f \in \mathcal {H}_{\lambda }$ be fixed. Define $\varphi = i (\nu _1 + i \nu _2) ( \gamma _D^+ f_{+} - \gamma _D^- f_{-}) \in H^{1/2}(\Sigma )$ and $g = \Psi _{\lambda } \varphi \in \mathcal {H}_{\lambda }$. By (iii) we have that

$$\begin{aligned} \begin{aligned} \gamma _D^+ ( f - g)_{+} - \gamma _D^- ( f - g)_{-}&= \gamma _D^+ f_{+} - \gamma _D^- f_{-} + i (\nu _1 - i \nu _2) \varphi = 0. \end{aligned} \end{aligned}$$

This shows $f - g \in H^1({\mathbb R}^2)$. Moreover, due to $f, g \in \mathcal {H}_{\lambda }$ we have that $\partial _{\overline{z}} (f - g) \in H^1({\mathbb R}^2)$, which implies $f-g \in H^2(\mathbb {R}^2)$. Combining this with $f, g \in \mathcal {H}_\lambda$ we find that $f-g \in \text {ker}\left( - \Delta - \lambda \right) = \{ 0 \}$, i.e. $f = g = \Psi _\lambda \varphi$. Thus $\Psi _{\lambda }: H^{1/2}(\Sigma ) \rightarrow \mathcal {H}_{\lambda }$ is also surjective and all claims in assertion (i) are shown. $\square$

### Proof of Proposition 2.2

The proof of item (i) is divided into 3 separate steps. In *Step 1* we show that the map $(-\infty , 0) \ni \lambda \mapsto \mu _n(S(\lambda )) \in (0, \infty )$ is continuous and strictly monotonically increasing, and in *Step 2* we verify that the same is true for the map $(-\infty , 0) \ni \lambda \mapsto \lambda \mu _n(S(\lambda )) \in (-\infty , 0)$. Using these results, we complete the proof of assertion (i) in *Step 3*.

*Step 1.* Let $n \in \mathbb {N}$. We show that the map $(-\infty , 0) \ni \lambda \mapsto \mu _n(S(\lambda )) \in (0,\infty )$ is continuous and strictly monotonically increasing. To verify that $\mu _n(S(\lambda ))>0$ for $\lambda \in (-\infty ,0)$, it suffices to prove that $S(\lambda )$ is a positive self-adjoint operator. From the definition of $S(\lambda )$ in (2.4) it follows that $S(\lambda )$ is self-adjoint. Next, let $\varphi \in L^2(\Sigma )$ with $\varphi \ne 0$ and set $f:= SL(\lambda ) \varphi$. Using the properties of $SL(\lambda )$ described at the beginning of this appendix one finds that $f \ne 0$ and

$$\begin{aligned} \begin{aligned} \big (S(\lambda ) \varphi&, \varphi \big )_{L^2(\Sigma )} = \big ( \gamma _D f , \partial _{\nu } f_{+} - \partial _{\nu } f_{-} \big )_{L^2(\Sigma )} \\&= \big ( f_{+} , \Delta f_{+} \big )_{L^2(\Omega _{+})} + \Vert \nabla f_{+} \Vert _{L^2(\Omega _{+})}^2 + \big ( f_{-} , \Delta f_{-} \big )_{L^2(\Omega _{-})} + \Vert \nabla f_{-} \Vert _{L^2(\Omega _{-})}^2 \\&\ge \big ( f_{+} , \Delta f_{+} \big )_{L^2(\Omega _{+})} + \big ( f_{-} , \Delta f_{-} \big )_{L^2(\Omega _{-})} = - \lambda \Vert f \Vert _{L^2({\mathbb R}^2)}^2 > 0. \end{aligned} \end{aligned}$$

Therefore, $\mu _n(S(\lambda )) > 0$ must be true.

Next, we show that $(-\infty , 0) \ni \lambda \mapsto \mu _n(S(\lambda )) \in (0,\infty )$ is monotonically increasing and continuous. With (A.2) one sees that $S(\cdot ): \mathbb {C} {\setminus } [0, \infty ) \rightarrow \mathcal {L}(L^2(\Sigma ))$ is holomorphic and that $\frac{d}{d \lambda } S(\lambda ) = \gamma _D (-\Delta - \lambda )^{-2} \gamma _D'$ holds. In particular, for any $\varphi \in L^2(\Sigma )$ the function $(- \infty , 0) \ni \lambda \mapsto (S(\lambda ) \varphi , \varphi )_{L^2(\Sigma )}$ is continuously differentiable and

$$\begin{aligned} \begin{aligned} \frac{d}{d \lambda } \big (S(\lambda ) \varphi , \varphi \big )_{L^2(\Sigma )}&= \big ( (-\Delta - \lambda )^{-1} \gamma _D' \varphi , (-\Delta - \lambda )^{-1} \gamma _D' \varphi \big )_{L^2(\Sigma )}\\ {}&= \Vert SL(\lambda ) \varphi \Vert _{L^2({\mathbb R}^2)}^2\ge 0 \end{aligned} \end{aligned}$$

is true. Thus, the min–max principle implies that the map $(- \infty , 0) \ni \lambda \mapsto \mu _n(S(\lambda ))$ is monotonically increasing for every $n \in {\mathbb N}$. Furthermore, due to the holomorphy of $S(\cdot ): \mathbb {C} {\setminus } [0, \infty ) \rightarrow \mathcal {L}(L^2(\Sigma ))$ and the estimate

$$\begin{aligned} | \mu _n(S(\eta )) - \mu _n(S(\lambda )) | \le \Vert S(\eta ) - S(\lambda ) \Vert ,\quad \eta ,\lambda <0, \end{aligned}$$

(see [29, Satz 3.17]), we find that $(- \infty , 0) \ni \lambda \mapsto \mu _n(S(\lambda ))$ is continuous for $n \in {\mathbb N}$.

It remains to show that the latter map is strictly monotonically increasing. Define for $\alpha \in {\mathbb R}{\setminus } \{0\}$ the operator-valued function $\mathcal {B}_1: {\mathbb C}{\setminus } [0,\infty ) \rightarrow \mathcal {L}(L^2(\Sigma ))$ by $\mathcal {B}_1(\lambda ) = I - \alpha S(\lambda )$. By the properties of $S(\lambda )$ it is easy to see that $\mathcal {B}_1$ is holomorphic and $\mathcal {B}_1(\lambda )$ is a Fredholm operator with index 0 for any fixed $\lambda$, since $S(\lambda )$ is compact in $L^2(\Sigma )$. Moreover, by [18, Theorem 1.2] there exists a constant $K>0$ such that

A.6 $$\begin{aligned} \Vert S(\lambda ) \Vert \le \frac{K}{ \sqrt{2+ |\lambda |}} \ln \sqrt{ 2 + \frac{1}{| \lambda |} } , \quad \lambda \in {\mathbb C}{\setminus } [0,\infty ). \end{aligned}$$

Hence, there exists $\lambda _0 < 0$ such that $\Vert S(\lambda ) \Vert < |\alpha |^{-1}$ is valid for all $\lambda < \lambda _0$. This implies that $\mathcal {B}_1(\lambda )$ is boundedly invertible for every $\lambda < \lambda _0$. Therefore, by [20, Chapter XI., Corollary 8.4] the set

$$\begin{aligned} \mathcal {M}_{\alpha ,1} = \left\{ \lambda \in {\mathbb C}{\setminus } [0, \infty ) \, \big | \, \mathcal {B}_1(\lambda ) = I - \alpha S(\lambda ) \text { is not invertible} \right\} \end{aligned}$$

is at most countable and does not have an accumulation point in ${\mathbb C}{\setminus } [0, \infty )$. Now assume that $\lambda _1< \lambda _2 < 0$ satisfy $\mu _n(S(\lambda _1)) = \mu _n(S(\lambda _2)) =: \alpha ^{-1}$ for some $n \in {\mathbb N}$. Then it follows from the monotonicity of $\lambda \mapsto \mu _n(S(\lambda ))$ that $[\lambda _1, \lambda _2] \subseteq \mathcal {M}_{\alpha ,1}$, which is a contradiction to the fact that $\mathcal {M}_{\alpha ,1}$ is at most countable. Therefore, the mapping $(- \infty ,0) \ni \lambda \mapsto \mu _n(S(\lambda ))$ is continuous and strictly monotonically increasing for $n \in {\mathbb N}$.

*Step 2.* To show the continuity and strict monotonicity of the map $(- \infty , 0) \ni \lambda \mapsto \lambda \mu _n(S(\lambda ))$ for all $n \in {\mathbb N}$, we note first that the continuity follows from the continuity of the map $\lambda \mapsto \mu _n(S(\lambda ))$ shown in *Step 1*. In order to prove the monotonicity, we use again $\frac{d}{d \lambda } S(\lambda ) = \gamma _D (-\Delta - \lambda )^{-2} \gamma _D'$ and compute for a fixed $\varphi \in L^2(\Sigma )$ and $\lambda \in (-\infty , 0)$ with the help of (2.5) and (A.2)

$$\begin{aligned} \begin{aligned} \frac{d}{d \lambda } \big ( \lambda S(\lambda ) \varphi , \varphi )_{L^2(\Sigma )}&= \big ( S(\lambda ) \varphi + \lambda \gamma _D (- \Delta - \lambda )^{-2} \gamma _D' \varphi , \varphi \big )_{L^2(\Sigma )} \\&= \big ( - 4 \gamma _D (-\Delta - \lambda )^{-1} \partial _{\overline{z}} \partial _{z} (- \Delta - \lambda )^{-1} \gamma _D' \varphi , \varphi \big )_{L^2(\Sigma )} \\&= \Vert \Psi _\lambda \varphi \Vert _{L^2({\mathbb R}^2)}^2 \ge 0. \end{aligned} \end{aligned}$$

Thus, the min-max principle yields the monotonicity of the mapping $(- \infty , 0) \ni \lambda \mapsto \lambda \mu _n(S(\lambda ))$. To see the strict monotonicity, we use a similar strategy as in *Step 1* and define for $\alpha \in \mathbb {R} {\setminus } \{ 0 \}$ the holomorphic mapping $\mathcal {B}_2: {\mathbb C}{\setminus } [0, \infty ) \rightarrow \mathcal {L}( L^2(\Sigma ))$ by $\mathcal {B}_2(\lambda ) = I - \alpha \lambda S(\lambda )$. Again, $\mathcal {B}_2(\lambda )$ is a Fredholm operator with index zero for any fixed $\lambda$ and it follows from (A.6) that there exists $\lambda _3 < 0$ such that $\Vert \lambda S(\lambda ) \Vert < |\alpha |^{-1}$ holds for all $\lambda \in (\lambda _3, 0)$. In particular, $\mathcal {B}_2(\lambda )$ is boundedly invertible for all $\lambda \in (\lambda _3, 0)$. It follows from [20, Chapter XI., Corollary 8.4] that the set

$$\begin{aligned} \mathcal {M}_{\alpha ,2} = \left\{ \lambda \in {\mathbb C}{\setminus } [0, \infty ) \, \big | \, \mathcal {B}_2(\lambda ) = I - \alpha \lambda S(\lambda ) \text { is not invertible} \right\} \end{aligned}$$

is at most countable and does not have an accumulation point in ${\mathbb C}{\setminus } [0, \infty )$. Now the same argument as in *Step 1* shows that $(- \infty , 0) \ni \lambda \mapsto \lambda \mu _n(S(\lambda ))$ is strictly monotonously increasing.

*Step 3.* To study the limiting behaviour of $\lambda \mu _n(S(\lambda ))$ for $\lambda \rightarrow 0$, note that (A.6) implies $\Vert \lambda S(\lambda )\Vert \rightarrow 0$ for $\lambda \rightarrow 0^-$ and hence,

A.7 $$\begin{aligned} \lim _{\lambda \rightarrow 0^{-}} \lambda \mu _n(S(\lambda )) = 0, \quad n \in {\mathbb N}. \end{aligned}$$

Next, we consider the limit of $\lambda \mu _n(S(\lambda ))$ for $\lambda \rightarrow -\infty$. For this purpose, results on Schrödinger operators with $\delta$-interactions will be used. Define for $\alpha < 0$ the sesquilinear form

$$\begin{aligned} \mathfrak h_{\delta , \alpha }[f, g] = \big ( \nabla f , \nabla g \big )_{L^2({\mathbb R}^2)} + \alpha \big ( \gamma _D f , \gamma _D g \big )_{L^2(\Sigma )}, \quad f, g \in \text {dom}\,\mathfrak h_{\delta , \alpha } = H^1({\mathbb R}^2). \end{aligned}$$

By [9, 14] the form $\mathfrak h_{\delta , \alpha }$ is semi-bounded and closed, and one can show for the self-adjoint operator $H_{\delta , \alpha }$, which is associated with $\mathfrak h_{\delta , \alpha }$ by the first representation theorem, that $\sigma _{\text {ess}}(H_{\delta , \alpha }) = [ 0, \infty )$, that its discrete spectrum $\sigma _{\text {disc}}(H_{\delta , \alpha })$ is finite, and for $\lambda \in (-\infty ,0)$ one has that

A.8 $$\begin{aligned} \lambda \in \sigma _p ( H_{\delta , \alpha }) \quad \Longleftrightarrow \quad -1 \in \sigma _p (\alpha S(\lambda )); \end{aligned}$$

see for instance [9, Lemma 2.3 and Theorem 4.2] and [8, Theorems 3.5 and 3.14]. Recall that the eigenvalues $\mu _n(S(\lambda ))$ are ordered non-increasingly with multiplicities taken into account. If we order the discrete eigenvalues of $H_{\delta , \alpha }$ in an increasing way then the strict monotonicity of $\lambda \mapsto \mu _n(S(\lambda ))$ implies that the *k*-th discrete eigenvalue $E_k(\alpha )$ (if it exists) satisfies the equation $-1 = \alpha \mu _k(S(E_k(\alpha )))$.

Let $n \in {\mathbb N}$. Then by [14, Theorem 1] the operator $H_{\delta , \alpha }$ has at least *n* negative discrete eigenvalues (counted with multiplicities) if $-\alpha >0$ is sufficiently large, and the *n*-th discrete eigenvalue $E_n(\alpha )$ of $H_{\delta , \alpha }$ admits the asymptotic expansion

A.9 $$\begin{aligned} E_n(\alpha ) = - \frac{\alpha ^2}{4} + \mu _n(H) + \mathcal {O}(\alpha ^{-1} \ln |\alpha |), \qquad \alpha \rightarrow - \infty . \end{aligned}$$

Here *H* is a fixed semibounded differential operator on $\Sigma$ that is independent of $\alpha$ and has purely discrete spectrum $\mu _1(H)\le \mu _2(H)\le \dots$. Thus for $\alpha \rightarrow -\infty$ we obtain with (A.8) that

A.10 $$\begin{aligned} \frac{\alpha }{4} + \frac{|\mu _n(H)|+1}{\alpha } \le E_n(\alpha ) \mu _n(S(E_n(\alpha ))) = -\frac{E_n(\alpha )}{\alpha } \le \frac{\alpha }{4} - \frac{|\mu _n(H)|+1}{\alpha }. \end{aligned}$$

This shows

A.11 $$\begin{aligned} \lim _{\lambda \rightarrow -\infty } \lambda \mu _n( S(\lambda ) ) = - \infty \end{aligned}$$

and finishes the proof of item (i).

To show item (ii), we note first that by (A.7), (A.11), and the strict monotonicity and continuity of the mapping $\lambda \mapsto \lambda \mu _n(S(\lambda ))$ it is clear that for any $a<0$ there is a unique solution $\lambda _n(a)$ of $\lambda \mu _n(S(\lambda )) = a$. Let $\mu _n(H)$ be as in (A.9), define the numbers $k_{\pm } = \pm ( |\mu _n(H)|+1 )$ and let

A.12 $$\begin{aligned} \alpha _{\pm } = -2 |a| \left( \sqrt{1 + \frac{k_{\pm }}{ a^2} }+ 1\right) = -4 |a| - \frac{k_{\pm }}{|a|} + f_{\pm }(a) \end{aligned}$$

with some functions $f_{\pm }(a) = \mathcal {O}( a^{-3} )$ for large $|a|>0$, where the latter representation holds due to a Taylor series expansion. Then one has

A.13 $$\begin{aligned} a = \frac{\alpha _{\pm }}{4} - \frac{k_{\pm }}{\alpha _{\pm }} \end{aligned}$$

and it follows with (A.10) that

$$\begin{aligned} E_n(\alpha _+) \mu _n(S(E_n(\alpha _+)))\le \frac{\alpha _+}{4} - \frac{|\mu _n(H)|+1}{\alpha _+}=a=\lambda _n(a) \mu _n ( S(\lambda _n(a)) \end{aligned}$$

and

$$\begin{aligned} \lambda _n(a) \mu _n ( S(\lambda _n(a)) = a = \frac{\alpha _{-}}{4} + \frac{|\mu _n(H)|+1}{\alpha _{-}} \le E_n(\alpha _-) \mu _n(S(E_n(\alpha _-))). \end{aligned}$$

Since $\lambda \mapsto \lambda \mu _n( S(\lambda ))$ is monotone we find

A.14 $$\begin{aligned} E_n( \alpha _{+})\le \lambda _n(a) \le E_n( \alpha _{-}). \end{aligned}$$

From (A.12) we obtain

$$\begin{aligned} \frac{1}{4} \alpha _\pm ^2=4 a^2 + 2 k_\pm + g_{\pm }(a) \end{aligned}$$

with functions $g_{\pm }(a) = \mathcal {O}( a^{-2} )$ for large $|a|>0$ and hence (A.9) implies

A.15 $$\begin{aligned} \big |E_n(\alpha _\pm ) + 4 a^2 + 2 k_\pm + g_{\pm }(a) - \mu _n(H)\big | \le C_1 \big |\alpha _\pm ^{-1} \ln |\alpha _\pm |\big | \end{aligned}$$

for some constant $C_1 >0$. Note that there exist positive constants $C_2, C_3 >0$ such that $C_2 \vert a \vert \le \vert \alpha _\pm \vert \le C_3 \vert a \vert$ holds for large $|a|>0$. With this we conclude from (A.15) that

A.16 $$\begin{aligned} \begin{aligned} \big |E_n(\alpha _\pm ) + 4 a^2 + 2 k_\pm - \mu _n(H)\big | \le C_4 \big | a^{-1} \ln |a| \big | \end{aligned} \end{aligned}$$

holds for some constant $C_4 > 0$ and for large $|a|>0$. Taking (A.14) and (A.16) into account, one concludes finally that

$$\begin{aligned} |\lambda _n(a) + 4a^2| \le 3 |\mu _n(H)| + 2 + \mathcal {O}(a^{-1} \ln |a|) = \mathcal {O}(1) \quad \text {for } a \rightarrow - \infty . \end{aligned}$$

$$\square $$
